# Report Made in the Name of the Committee on Mineral Waters for 1837

**Published:** 1839-06-29

**Authors:** Ph. Patissier


MEDICAL EXAMINER.

DEVOTED TO MEDICINE, SURGERY, AND THE COLLATERAL SCIENCES.

No. 26.] PHILADELPHIA, SATURDAY, JUNE 29, 1839. [Vol. II.

*Report made in the name of the Committee on Mineral Waters for* 1837. By M. Ph. Patissier.

(Continued from page 394.)

*Section* 4.—Of chronic diseases which may be developed in all parts of the body.

*Chronic Rheumatic Affections, Lumbago.*—These diseases are so common that they affect three-fourths of the persons who visit thermal establishments; and it is in the treatment of them that warm mineral waters are most efficacious. It is observed by practising physicians that baths of common water, taken at an elevated temperature, are not adapted for the cure of rheumatisms; for such baths, by diminishing in a great degree the energy of the skin, render it peculiarly sensible to the impression of cold and atmospheric moisture; whereas mineral baths, by stimulating the cutaneous system, enable it to react against atmospheric influences. From the fact that many rheumatic persons return annually to warm springs, it has been concluded by some, that in place of curing radically rheumatic affections, they only allay them for a longer or shorter time; but when a patient is cured of a rheumatism, he is not at all exempt from contracting a new one; the same causes may produce the same effects, and the latter bring back the patient to the means of cure or relief which had formerly succeeded with him. The best proof of the powerful action of thermal waters in cases of chronic rheumatisms, is the great number of the patients who resort to them annually, for a remedy soon ceases to be employed which fails to cure. Among the great number of persons who are affected with rheumatisms, and who go to the springs, some are cured, and others are relieved to a greater or less degree, provided they take the precaution of furnishing themselves with warm clothing, and wearing flannel next to the skin, which has the double advantage of preventing taking cold, and of keeping up the excitement produced upon the skin by the use of the baths. It should not be forgotten, however, that during, and often a short time after the use of warm mineral baths, the rheumatic pains are exasperated or renewed; and that it is a bad sign if these pains are not calmed during the immersion, and that, in this case, the disease is either complicated with the venereal affection, or symptomatic pains of a morbid condition of the viscera, or of the deep lesion of some articulation, have been taken for rheumatic pains, (M. Bertrand.) The emaciation of the limbs, which is the result of long rheumatic pains, does not contraindicate the use of mineral baths, but it renders the cure longer and more difficult.

We must not think, however, that all warm springs are equally efficacious in the treatment of all kinds of rheumatism; those which are of long standing, and which affect persons who are robust and difficult of receiving impressions, are generally lessened in their intensity or cured, promptly, by the use of the waters of Mont d'Or, Bareges, Bourbonne, Bourbon-l'Archambault, Balaruc, Bagnères de Luchon, &c., taken in warm baths, strong, warm douches, and vapour baths; but if the rheumatism is recent, and accompanied by inflammation,—if the patient is of great nervous susceptibility, the mild waters of Neris, Bains, Bagnères de Bigorre, Plombieres, &c., should be used in baths of moderate warmth, and in shower douches. The sulphur springs, particularly those of Barèges, are of less service in gouty or arthritic rheumatisms than in muscular rheumatisms. A gendarme, who had had several attacks of gouty rheumatism, was taken, after the fifteenth bath at Barèges, with dreadful pains in the joints, which yielded in the end only to adoucissans, to repose, and to the warmth of the bed. M. Gax, who cites this fact, reports that he himself was threatened with a new attack of articular rheumatism, to which he had been subject before, when, in order to become well acquainted with their action, he tried the waters of Barèges; after the fifth or sixth bath he felt in the thumb of the left hand a very intense pain, which went off on promptly abandoning the use of the waters. The waters of Mont d'Or, Vichy, Bourbon-l'Archambault, &c., are employed with success in gouty rheumatism without inflammation, attacking persons of but slightly irritable temperaments. It is particularly in the treatment of this kind of rheumatism that the waters of Bourbonne are so very efficacious. "Several patients affected with this disease," says M. Therreau in his report, "after having in vain tried the different kinds of treatment indicated under like circumstances, came to Bourbonne still using crutches, which they had not been able to dispense with for many years past, and went away without them, either completely cured, or in a fair way of being so." "Few patients," says M. Bertrand, "find a more rapid relief at Mont d'Or, than those who have gouty rheumatism; the crutches left at this watering place after the cures of such diseases, would be numerous, had there been a place where they might have been deposited, and each year would increase the collection."

STATISTICAL TABLE.

| NAMES OF DISEASES. | *Name of establishment.* | *Number of each disease.* | *Number of patients cured.* | *Number of patients relieved.* | *Number of patients treated without success* | *Number of patients cured or relieved after leaving the watering place.* |
|---|---|---|---|---|---|---|
| Muscular rheumatism. | Bourbonne. | 118 | 51 | 54 | 13 | 0 |
| Arthritic rheumatism. | Id. | 66 | 25 | 37 | 4 | 0 |
| Muscular rheumatism. | Barèges. | 98 | 56 | 30 | 12 | 0 |
| Lumbago. | Id. | 6 | 3 | 3 | 0 | 0 |
| Muscular rheumatism. | Rennes, (Aude.) | 110 | 15 | 35 | 60 | 0 |
| Rheumatisms. | Greoulx. | 83 | 5 | 48 | 30 | 24 |
| Lumbago. | Id. | 27 | 2 | 19 | 6 | 8 |
| Rheumatisms. | Bagnols, (Lozere.) | 459 | 156 | 199 | 104 | 182 |
| Muscular rheumatism. | Bourbon-l'Archambault.* | 957 | 480 | 400 | 77 | 0 |
| Articular rheumatism. | Id. | 850 | 415 | 425 | 10 | 0 |
| Muscular rheumatism. | Neris. | 26 | 2 | 20 | 4 | 10 |
| Articular rheumatism. | Id. | 24 | 2 | 19 | 3 | 9 |
| Nervous rheumatism. | Id. | 30 | 0 | 25 | 5 | 9 |
| Nervous rheumatism. | Bains. | 37 | 12 | 18 | 7 | 8 |
| Articular rheumatism. | Id. | 16 | 3 | 13 | 0 | 0 |

*Gout.*—This disease, which is called the sister of rheumatism, differs from it, however, in several points; rheumatism is usually caused by the suppression of transpiration, while gout (filia veneris et Bacchi) is most frequently produced by an excess in sexual gratification, by the immoderate use of spirituous liquors, and by a succulent nourishment taken in larger quantities than the wants of the body require. Baths of warm mineral waters, by provoking abundant sweats, are of great efficacy in the treatment of rheumatism. They are, on the contrary, injurious, especially sulphur waters, in gout; we must except, however, the baths of Neris, which ameliorate the condition of gouty persons during a year or two, and especially the waters of Vichy, which seem to be endowed with the precious privilege of curing the gout, or, at least, putting off for a long time its attacks, as is proved by several observations published by M. Charles Petit. The frequent coexistence of gout with gravel has created the belief that the fluids of gouty persons contain uric acid, which has, in fact, been detected in them by modern chemists; and it is this belief which induced M. Petit to employ the waters of Vichy in the treatment of this, previously reputed, incurable disease. These waters, however, only succeed when the patient changes his diet and his habits, remains sober, gives up the exclusive use of meats and other aliments containing azote, abstains from pure wine and alcoholic liqours, and submits himself to the frequent, if not habitual, use of the waters of Vichy, or at least of common water rendered more or less alkaline by means of bicarbonate of soda. Some practising physicians, and even M. Prunelle, the titular physician of Vichy, agree with the late M. Lucas in the opinion that the waters of Vichy, of which he had been inspector for thirty years, were more injurious than serviceable in gout. Alkalies have been, however, for a long time recommended in the treatment of gout: "they may be used with advantage," says M. Desbois of Rochefort, "in gout and rheumatism, when these affections are not of an inflammatory character, or when they are of long standing, with nodes and old gouty concretions; which articular concretions they are even of considerable efficacy in resolving." But the alkaline salts, such as bicarbonate of soda which abounds in the waters of Vichy, are much better borne by the stomach than the pure alkalies, and can be taken in the largest doses without inconvenience. Facts, moreover, are more to be depended upon than theory, and it appears to us impossible to contest those which have been related by M. Petit in two little works which he has addressed to you, and which show that under the influence of the waters of Vichy, several gouty persons have either been completely cured, or obtained a notable alleviation of their sufferings. In support of these facts, your reporter can cite the history of one of his near relations, aged fifty-four years, and gouty by inheritance for the last fifteen years; his legs habitually swollen, and his feet often painful, forced him to use a cane in walking.

* From 1824 to 1833.

It is hardly necessary to say that this patient had used, without success, all the remedies against gout, praised by charlatanism. In 1837 he went to Vichy, where he used the waters internally, and in baths, for the space of a month. Six weeks after his return home, he wrote to me that he could walk two leagues without the assistance of a cane, and without fatigue. Having drank from time to time, during the winter and spring, alkaline water, and having observed a rigid diet, which he even adhered to before using the waters, he has since had no attack of gout. In the month of March, however, he complained of pains about the bladder, and as it was possible that these pains might depend upon the retrocession of the gout upon this viscus, which retrocession had taken place several years before, he went again to Vichy in the month of July, 1838, and is so much improved in health from this second trip, that he now actually walks up mountains without difficulty.

Notwithstanding the opinion of M. Charles Petit, that patients with gout and stone in the bladder can take the waters of Vichy in large doses, without inconvenience, we think that generally these very active waters should be drank with reserve, as without this precaution they may produce gastritis and acute hepatitis. These inflammations, it appears to us, are much more to be feared than the solution of the fibrine of the blood by an excess of soda, and the bloody effusions which ought, according to M. Magendie, to result from this solution, effects which clinical observation has not demonstrated up to this day.

*Paralyses.*—Mineral springs of an elevated temperature are very efficacious in the treatment of paralyses, which have for their cause a rheumatismal or herpetic metastasis, or which are the consequence of internal diseases, or produced by metallic emanations; sulphur waters appear to have a special action against paralysis caused by lead. In paralysis, resulting from rheumatism, it is a good sign if the pains which preceded this affection, are revived during the use of the baths, or if the skin is affected with heat, local redness, or an eruption of small pimples.

STATISTICAL TABLE.

| NAMES OF DISEASES. | *Name of establishment.* | *Number of each disease.* | *Number of patients cured.* | *Number of patients relieved.* | *Number of patients treated without success* | *Number of patients cured or relieved after leaving the watering place.* |
|---|---|---|---|---|---|---|
| Different paralyses. | Bourbonne. | 25 | 6 | 12 | 7 | 0 |
| Different paralyses. | Bourbon-l'Archambault.* | 310 | 91 | 200 | 19 | 0 |
| Different paralyses. | Balaruc. | 5 | 2 | 3 | 0 | 0 |
| Rheumatic paralyses. | Rennes, (Aude.) | 34 | 4 | 10 | 20 | 5 |
| Different paralyses. | Neris. | 23 | 0 | 17 | 6 | 4 |
| Rheumatic hemiplegia. | Mont d'Or. | 11 | 2 | 5 | 4 | 1 |
| Different paralyses. | Bagnols, (Lozere.) | 54 | 10 | 21 | 23 | 19 |
| Paralyses of inferior limbs. | Barèges. | 5 | 4 | 1 | 0 | 0 |

*Chronic diseases of the skin.*—It is generally agreed that, notwithstanding the labours of modern pathologists, diseases of the skin are very difficult to cure; we know, also, that patients affected with diseases of this kind, abound at mineral springs. Sulphur waters, particularly those of Barèges, Bagnères de Luchon, &c., are certainly very useful in the treatment of these diseases; but their use is abused; they are only suitable when the exanthemata are of long standing, without inflammation, without complication with venereal affections, and when they attack individuals of lymphatic temperaments. It is under such circumstances that the waters of Bourbonne, Bourbon-l'Archambault, Mont d'Or, and sea baths succeed sometimes. One of the most constant results of these baths, is to increase, at first, the eruption; but this should cause no alarm, for it is seen to diminish shortly afterwards in a remarkable manner. But when cutaneous diseases affect persons of irritable temperaments, and when they are accompanied by a greater or less inflammation of the skin, baths of the slightly saline waters of Saint Alban, Avenes, Neris, Plombieres, Bagnoles (Orne,) Lonesche, &c., are more beneficial, and they are most so when they are taken of a moderate warmth, when they are prolonged, and when the skin is allowed to be macerated, as it were, in the warm water. It is probably to the unctuous quality of these waters, and to the smallness of the quantity of mineral principles which they contain, that they owe their property of softening the integuments, and of appeasing the irritations of which they are the seat.

Out of one hundred and twelve soldiers affected with herpetic diseases, and treated in 1829 at Barèges, by our colleague, M. Gax, the proportion cured was 31 in 51, for the simple herpetic affections (without designation;) in pustulous herpes* the proportion was a half, that is to say 10 in 20; in scaly herpes it was 14 in 18; in squamous herpes, 7 in 10; in inflammatory her-

*From 1824 to 1833.

*Herpes is the term used by Alibert for all scaly eruptions.

pes, 1 in 3; in syphilitic herpes, 1 in 5; in meulagra, 1 in 2; the only case of psoric herpes was cured; a repercessed herpes obtained but a slight relief. The waters of Avenes (Herault) seem, according to the report of their inspecting physician, to exert a special action in cases of acute herpes, eczema, and the pustular varieties of herpes; their action is less efficient in the scaly varieties; it is powerful in repercussions of the itch.

### STATISTICAL TABLE.

| NAMES OF DISEASES. | *Name of establishment.* | *Number of each disease.* | *Number of patients cured.* | *Number of patients relieved.* | *Number of patients treated without success.* | *Number of patients cured or relieved after leaving the watering place.* |
|---|---|---|---|---|---|---|
| Herpetic affections. | Bagnères de Luchon. | 68 | 24 | 37 | 7 | 0 |
| Id. | Bagnols, (Lozere.) | 96 | 21 | 39 | 36 | 0 |
| Id. | Greoulx. | 64 | 14 | 42 | 8 | 0 |
| Id. | Bourbon-l'Archambault. | 210 | 36 | 174 | 0 | 0 |
| Id. | Bourbonne. | 61 | 14 | 29 | 18 | 0 |
| Id. | Mont d'Or. | 19 | 6 | 7 | 6 | 6 |
| Id. | Neris. | 7 | 0 | 6 | 1 | 0 |
| Id. | Bains. | 4 | 1 | 3 | 0 | 0 |

The collectors of these statistics have the frankness to confess, that among the patients whom they put down as cured, there may be a great number in whom the cure is only apparent; for in order to know with certainty what has been the result of their treatment, we must be able to have the patients under our observation for a long time after they have left the watering place, and see them again, one, two and three years after we believed them cured. It is thus, only, that we can assure ourselves that the cure has been real and permanent; for we know that herpetic diseases are so apt to make their appearance again after being apparently cured, that none other can be more obstinate under treatment. "We must mistrust, says Borden, "those remedies which promptly diminish, or cause to disappear, herpetic eruptions; for these too quick changes, threaten the viscera with some dangerous affection."

*Syphilitic affections.*—All warm mineral waters, on account of their sudorific effects, contribute to the development of secondary syphilitic affections, when the venereal virus is still latent; hence they may be used when its existence is inspected. Sulphur baths assist very much in the mercurial treatment, and a remarkable effect of the combined treatment is the absence of all salivation, notwithstanding the introduction of considerable doses of mercury into the economy, depending, no doubt, upon the abundant perspiration produced by the use of the sulphur waters. These waters repair, also, the damages done by mercury administered without caution; drank and used as gargles, they cicatrize the ulcers in the mouth and on the velum palati, fix the loose teeth, and by restoring to the stomach and intestines their lost energy, regain to the patient his strength and flesh.

The object of treatment in all scrofulous affections, being to make the sanguineous system predominate over the lymphatic, it is readily conceived that sulphur and chalybeate mineral waters, and sea-baths, by reason of their tonic and stimulating properties, are adapted to fulfil perfectly this indication. Under the influence of these waters, therefore, we see scrofulous children acquire a more animated complexion, their digestion become regular, their strength become developed, and their abdomens, if large and indurated, become soft and return to their normal condition; the wounds and fistulous orifices which are made by the opening of abscesses, assume a florid colour, and take on all the characters of healthy wounds. It is, nevertheless, rarely that mineral waters, when employed alone, cure scrofula. "I know not by what fatality it is," says Borden, "that I have so seldom seen tumours and swollen glands completely discussed and removed by the waters of Pyrenèes; and this after a rigid observation." Out of thirteen scrofulous patients whom M. Gax treated at Barèges, one only, who had an engorgement of the glands of the neck, and a chronic ophthalmia, was cured. To increase the power of the waters, of Barèges, Borden was in the habit of associating with their use, mercurial frictions upon the engorged glands and ulcers; the efficacy of iodine in the treatment of scrofula having since been discovered, it may now be substituted for mercury.

STATISTICAL TABLE.

| NAMES OF DISEASES. | *Name of establishment.* | *Number of each disease.* | *Number of patients cured.* | *Number of patients relieved.* | *Number of patients treated without success.* | *Number of patients cured or relieved after leaving the watering place.* |
|---|---|---|---|---|---|---|
| Scrofulous engorgements. | Mont d'Or. | 12 | 0 | 5 | 7 | 0 |
| Engorgements of the submaxillary glands. | Id. | 7 | 3 | 1 | 3 | 0 |
| Scrofulous affections. | Balaruc. | 13 | 0 | 5 | 8 | 0 |
| Engorgements of lymphatic glands, abscesses, ulcers, fistulous openings, &c. | Bourbonne. | 132 | 57 | 62 | 13 | 0 |
| Scrofulous ulcers. | Bourbon-l'Archambault. | 43 | 18 | 15 | 10 | 0 |
| Scrofula. | Neris. | 4 | 0 | 2 | 2 | 1 |
| Scrofula. | Bagnoles, (Lozere.) | 78 | 17 | 38 | 23 | 40 |
| Scrofulous affections. | Bagnères, (Luchon.) | 41 | 14 | 10 | 17 | 9 |
| Scrofulous affections. | Sea-baths, Boulogne. | 9 | 3 | 6 | 0 | 0 |

We know that *Rachitis* acts upon the osseous system, alters its structure, softens it, and produces a great number of deformities, either in the vertebral column, or in the limbs. Sea baths, sulphur and chalybeate waters, by exercising a tonic effect upon the whole body, strengthen the limbs, and give to the bony structure a sufficient development and hardness to enable it to preserve its natural form, or to resist all farther deformity. In cases of this disease, the mild waters of Ussat, Neris, Bains, &c. should not be used, as they have the property of softening the fibrous tissue, the fibro-cartilages, and even the bony tissue in a remarkable degree.

Section V. *Surgical diseases.*—It remains for us to speak of stiffness of joints, of muscular retractions, and muscular weakness, of articular swellings, incomplete anchyloses, &c.; all the thermal waters are, in these cases, of pretty nearly the same efficacy. We must not, however, forget that patients convalescent from fractures, should not be sent to warm mineral springs, until six months after the accident, when the callus is perfectly solidified, for several of these springs, and particularly those of Bourbonne, Neris, and Carlsbad, possess the singular property of softening bony tissue, and disposing the callus to rupture; an important remark, and one from which useful hints may be drawn for the straightening of fractured limbs which have been improperly set. As to gun-shot wounds, fistulous ulcers, and caries, these diseases are more surely cured by the waters of Barèges, Bagnères de Luchon, Bains near Arles, Bourbonne-les-Bains, Bourbon-l'Archambault, &c., and by sea baths, than by other warm mineral springs.

STATISTICAL TABLE.

| NAMES OF DISEASES. | *Name of establishment.* | *Number of each disease.* | *Number of patients cured.* | *Number of patients relieved.* | *Number of patients treated without success.* | *Number of patients cured or relieved after leaving the watering place.* |
|---|---|---|---|---|---|---|
| Hydarthroses. | Mont d'Or. | 8 | 1 | 3 | 4 | 2 |
| Caries of vertebræ. | Id. | 2 | 0 | 0 | 2 | 0 |
| Secondary luxations, threatened or completed. | Id. | 10 | 0 | 4 | 6 | 0 |
| White swellings, from metastasis of rheumatism. | Rennes, (Aude.) | 42 | 8 | 14 | 20 | 9 |
| White swelling, with exostosis. | Id. | 31 | 0 | 6 | 25 | 2 |
| Chronic sprains. | Id. | 74 | 4 | 30 | 40 | 3 |
| Gunshot wounds, fistulous wounds. | Barèges. | 8 | 2 | 6 | 2 | 0 |
| Pains in bones. | Id. | 5 | 0 | 2 | 3 | 0 |
| Articular engorgements. | Id. | 12 | 3 | 3 | 6 | 0 |
| False anchyloses. | Id. | 9 | 0 | 6 | 3 | 0 |
| Muscular retraction. | Id. | 3 | 0 | 2 | 1 | 0 |
| Lesions consequent upon gunshot wounds, and on wounds by cutting instruments. | Bourbonne. | 48 | 24 | 22 | 2 | 0 |
| Id. | Bains-d'Arles. | 27 | 7 | 16 | 4 | 0 |

We have summed up the preceding considerations in the following table, which shows at a glance what waters are best adapted to the treatment of each disease:

TABLE *of the most frequent Chronic Diseases, with a designation of the Mineral Waters adapted to their treatment.*

| | *Names of diseases.* | *Indications for treatment.* | *Designation of mineral waters adapted to the disease.* |
|---|---|---|---|
| Diseases of the head. | Paralysis, (following apoplexy.) | When there exists no signs of congestion in the brain, and when the patient is of a lymphatic or little irritable temperament. | Waters of Bourbonne, Balaruc, Bourbon-l'Archambault, taken internally, in tempered baths, and douches upon the paralysed part. |
| | Nervous affections, hysteria, hypochondria, catalepsy, chorea. | If these maladies are recent, idiopathic, without complication. | Waters of Ussat, of St. Sauveur, of Salut at Bagneres de Bigorre, of Neris, Bains, &c., taken internally, in tempered baths, and in shower douches. |
| | Facial neuralgia, (tic douloreux.) | | Same waters taken internally, in baths, and Scotch douches upon the head; sea-baths, with affusion. |
| | Goitre. | If it depends upon hypertrophy of the thyroid gland. | Waters of Heilbrunn in Bavaria, internally, and in douches. |
| Diseases of the chest. | Chronic pulmonary catarrh, chronic pneumonia, chronic pleurisy, laryngeal phthisis, pulmonary phthisis in first degree, idiopathic humid asthma, passive hæmoptysis. *Note.*—These diseases are curable by the waters when there is no fever, heat, or dryness of skin, and when their cause is metastatic.* | If the patient is of a lymphatic, or little irritable constitution. | Waters of Mont d'Or, of Bonnes, of Raillère at Canterets, &c., internally, and in semicupia. |
| | | If the patient is of a dry, nervous constitution. | Waters of Ems, (Duchy of Nassau.) |
| | Palpitations. | If they depend upon general atony and chlorosis; if they are nervous. | Chalybeate waters of Forges, Spa, Piedmont, &c.; sulphur waters internally, and in baths. |
| | Aneurism of the heart or large arteries. | All mineral waters do injury, by quickening the circulation. | |
| Diseases of the abdomen, (digestive canal.) | Chronic gastritis, chronic enteritis, gastralgia, nervous vomiting, anorexia, flatulence, chronic diarrhœa. *Note.*—These diseases are only curable by mineral waters when they are not caused by scirrhous or cancerous affections. | When these diseases are the result of inflammation, or of a nervous character. | Cold acidulated waters of Ponges, Chateldon, Seltz, Contrexville, &c.; waters of Plombieres, taken internally, and in baths. |
| | | If there is atony of the digestive canal. | Chalybeate waters of Forges, Spa, &c.; sulphur waters of Canterets, &c., internally, and in baths. |
| | | If these diseases are owing to a bilious or mucous state of the gastro-intestinal canal. | Waters of Niederbronn, of Balaruc, and Bagneres de Bigorre, internally. |
| | | If these maladies are the result of a retrocession of a morbid principle. | Warm mineral waters, internally, in baths, douches, and vapour baths. |
| | Mesenteric atrophy, (tabes mesenterica.) | | Chalybeate waters; sea-baths. |

* That is, depending upon the suppression of the transpiration of a habitual flux, or the retrocession of rheumatismal, gouty, herpetic, and psoric principles.

*Same Table continued.*

| | *Names of diseases.* | *Indications for treatment.* | *Designation of mineral waters adapted to disease.* |
|---|---|---|---|
| Diseases of the digestive canal. | Engorgement of the abdominal viscera, (obstructions of the liver and spleen,) biliary calculi, jaundice, old intermittent fever. *Note.*—Mineral waters can remove the visceral engorgements when they are recent, passive, and the product of a congestion of blood, or of a simple hypertrophy of the liver or spleen. | If the patient is of a lymphatic constitution, and if there exists no traces of inflammation. | Waters of Vichy; chalybeate waters internally, in baths, and in douches upon the abdomen. |
| | | If the disease is nervous; if the irritation of the viscus is not entirely removed. | Cold, acidulated waters, (Ponges, Contrexville, Seltz, Chateldon, &c.,) internally; waters of Plombieres. |
| | | If the digestive canal is in a bilious or mucous state. | Laxative waters of Niederbronn and Balaruc; purgative waters of Seidlitz, Pullna, &c. |
| | | If the engorgement is caused by metastasis. | Warm mineral waters internally, in baths, in douches and steam-baths. |
| | Hæmorrhoidal flux. | If the flux is passive and abundant. | Chalybeate waters internally. |
| | | If the flux is suppressed. | Warm mineral waters internally, in baths and douches directed towards the rectum. |
| Diseases of the urinary passages. | Incontinence of urine. | If it is the result of a general or local feebleness. | Sea-baths; sulphur waters internally; baths slightly cold, and douches upon the lumbar region. |
| | Chronic vesical catarrh. | If there exists any signs of irritation; if the disease is nervous. | Cold, acidulated waters, as of Ponges, Contrexville, Seltz, &c., internally. |
| | Chronic catarrh of the bladder. | If the patient is of a lymphatic constitution, and if there exists no traces of inflammation. | Waters of Vichy, St. Nectaire, and Mont d'Or; sulphur waters internally, in baths, and in douches. |
| | | If the disease is the result of a retrocession. | Warm mineral waters internally, in baths, douches, and steam-baths. |
| | Gravel. | | Waters of Vichy, St. Nectaire, Contrexville; all cold, acidulated waters internally, and in baths. |
| | Urinary calculi. | | The waters of Vichy, which are rich in bicarbonate of soda, are the only waters which clinical observation has proved, up to this day, to be adapted to the solution of urinary calculi. |
| Diseases of the genital organs. Male. | Impotency, exhaustion resulting from masturbation, or excess in sensual pleasures; involuntary seminal discharges; blenorrhœa. | | Sulphur waters; saline waters of Bourbonne, Balaruc; waters of Mont d'Or, Bourbon-l'Archambault, &c., taken internally, and in moderately warm baths and douches upon the lumbar region; sea-baths in the waves. |
| Female. | Amenorrhœa and dysmenorrhœa. | If the suppression of the menses is owing to atony, as with chlorotic females. | Sulphur and chalybeate waters internally, and in baths and douches; sea-baths. |
| | | If the suppression of the menses is produced by plethora, or by an excess of sensibility of the uterus. | After the antiphlogistic treatment, the waters of Neris, Luxeuil, Bains, &c., in moderately warm baths. |
| | Menorrhagia. | If it is passive without organic lesion. | Sulphur waters and chalybeates; sea-baths in the waves. |

*Same Table continued.*

| | *Names of diseases.* | *Indications for treatment.* | *Designation of mineral waters adapted to the disease.* |
|---|---|---|---|
| Diseases of the Genital Organs. Female. | Leucorrhœa. | When it is owing to a general or local feebleness. | Sulphur and chalybeate waters, and saline waters of Bourbonne, Balaruc and Mont d'Or, internally, and in baths and douches directed towards the vagina; sea-baths. |
| Diseases of the Genital Organs. Female. | Relaxation or prolapsus of the uterus. | | Same treatment, especially sea-bathing. |
| Diseases of the Genital Organs. Female. | Chronic metritis. | When the patient is of a lymphatic constitution, and there exists no farther inflammation. | Same treatment; douches directed towards the vagina. |
| Diseases of the Genital Organs. Female. | Chronic metritis. | If there still exists a little phlogosis, and if the patient is of a dry, nervous constitution. | Cold acidulated waters of Neris, Luxeuil, Bains, &c., internally, and in baths of moderate warmth. |
| Diseases of the Genital Organs. Female. | Sterility. | If the sterility can be attributed to a feeble, or to a too abundant leucorrhœal flow, or to a want of excitability in the uterus. | Sulphur and chalybeate waters; waters of Bourbonne, of Balaruc, and Mont d'Or, internally, in baths and douches; sea baths. |
| Diseases of the Genital Organs. Female. | Sterility. | If the sterility is owing to a nervous affection, or to an excess of general or local sensibility. | Waters of Neris, St. Sauveur, Bagnères de Bigorre, Plombieres, Bourbon-Laucy, &c.; internally and in baths of moderate warmth. |
| Diseases which may affect every part of the body. | Rheumatic affections, (lumbago and sciatica.) | If the rheumatism is of long standing, and if it affects a robust individual, difficult of receiving impressions. | Sulphur waters; waters of Balaruc, Mont d'Or, &c., internally, in baths, douches, and steam baths. |
| Diseases which may affect every part of the body. | Rheumatic affections, (lumbago and sciatica.) | If the rheumatism is recent, and accompanied by a great sensibility. | Waters of Neris, Plombieres, Luxeuil, Bagnères de Bigorre, &c., in baths of moderate warmth, and shower douches. |
| Diseases which may affect every part of the body. | Chronic gout. | In the intervals of the attacks. | Waters of Neris, and especially of Vichy, internally, and in baths. |
| Diseases which may affect every part of the body. | Paralysis without cerebral lesion. | If it is caused by a metallic emanation. | Sulphur waters internally, and in warm baths and douches. |
| Diseases which may affect every part of the body. | Paralysis without cerebral lesion. | If the cause is metastatic. | All thermal waters internally, in warm baths and douches. |
| Diseases which may affect every part of the body. | Chronic diseases of the the skin, (herpes, *conperose*, *ephélide*, old itch, and disposition to erysipelas, or boils.) | When there is no acute inflammation of the skin and the patient is of a soft, lymphatic temperament. | Sulphur waters of Bareges, Bagnere de Luchon, Molitg, &c., internally, in baths, douches, and steam baths. |
| Diseases which may affect every part of the body. | Chronic diseases of the the skin, (herpes, *conperose*, *ephélide*, old itch, and disposition to erysipelas, or boils.) | If there exists a strong irritation in the skin, and if the patient is of an irritable constitution. | The slightly saline waters of Avenes, Neris, Plombieres, Bagnoles, (Orne,) Luxeuil, Lonesche, St. Gervais, &c., internally, in moderately warm baths, prolonged, and in shower douches. |
| Diseases which may affect every part of the body. | Scrofula; engorgements of glands; strumous ulcers; strumous ophthalmia; rachitis. | If there exists no symptoms of inflammation. | Sulphur and chalybeate waters; saline waters of Bourbonne and Balaruc; the waters of Mont d'Or; internally, in baths and douches; sea baths. |
| Diseases which may affect every part of the body. | Syphilitic diseases; secondary syphilis; mercurial cachexia. | | All thermal waters, internally and in baths, contribute to the development of venereal diseases when they are still latent; sulphur waters aid the mercurial treatment and repair the damages effected by mercury administered without circumspection. |

*Same Table continued.*

| | *Names of diseases.* | *Indications for treatment.* | *Designation of mineral waters adapted to the disease.* |
|---|---|---|---|
| Diseases which may affect every part of the body. | General debility; stiffening of limbs; feeling of cold in a limb, accompanied by muscular weakness. | | Sulphur waters, waters of Bourbonne, Balaruc, Mont d'Or, Bourbon-l'Archambault, &c., in baths slightly warm, and warm douches upon the stiffened parts, and the vertebral column; sea baths. |
| Surgical Diseases. | Stiffness and contraction of limbs after fractures; luxations, sprains or contusions; emaciation, commencing atrophy of members; hydarthroses with complete anchylosis. | | Muds of Saint Armaud, de Barbotan, and all warm springs. Convalescents from fractures should not resort to them for six months after the consolidation of the callus, as several of these springs have the property of softening bony tissue, and new fractures might occur. |
| | White-swelling. | When the white-swelling is rheumatic in its nature without inflammation. | Most thermal waters in baths and shower douches. |
| | | If the engorgement is articular, or is maintained by a scrofulous diathesis. | Sulphur and chalybeate waters, internally in baths and douches. |
| | Accidents resulting from gun-shot wounds, fistulous ulcers, caries of bones. | | Waters of Bareges, Aix-in-Savoy, Bagneres, de Luchon, Bourbonne, Balaruc, Bourbon-l'Archambault, &c., in baths, and especially in douches. |

The details into which we have been entering, go to show that mineral waters constitute an active remedial agent, which, when prescribed properly, is a resource of great value in the treatment of many chronic diseases, for the cure of which ordinary means are powerless; that their employment ought to be regulated by the general rules of therapeutics, that is to say, by the indications to be fulfilled; that diseases similar in appearance, require the use of different mineral waters; in a word, that to fix upon the choice of a mineral water, the physician ought to take into consideration the different circumstances of the disease, its causes, its periods, its complications, and the temperament of the patient.

Although we have made known to you the results of but a few of the recapitulative tables sent by the inspecting physicians, still, what we have said suffices to show you that if these tables are made with care and good faith, the Academy may, in a few years, make up a well grounded opinion upon the medicinal effects of the mineral waters of France; compare them with one another as to their action in diseases of nearly the same nature, and by considering this action in connection with the constituent elements of the waters, substitute rational principles for the motives, for the most part empirical, which influence physicians, at present, in their employment of them. Unfortunately, the clinical facts which serve as a base for these tables, being unprovided with particulars upon the causes and symptoms of the diseases, are not of great scientific value. The Academy, on this account, gives notice to the inspecting physicians, that it will highly approve of the zeal of those who will join to their reports special observations well detailed, and all other documents which may serve to throw light upon the therapeutical properties peculiar to each mineral spring.

